# Analysing Syntactic Regularities and Irregularities in SNOMED-CT

**DOI:** 10.1186/2041-1480-3-8

**Published:** 2012-12-17

**Authors:** Eleni Mikroyannidi, Robert Stevens, Luigi Iannone, Alan Rector

**Affiliations:** 1 School of Computer Science, The University of Manchester, Oxford Road, Manchester, M13 9PL UK

## Abstract

**Motivation:**

In this paper we demonstrate the usage of RIO; a framework for detecting syntactic regularities using cluster analysis of the entities in the signature of an ontology. Quality assurance in ontologies is vital for their use in real applications, as well as a complex and difficult task. It is also important to have such methods and tools when the ontology lacks documentation and the user cannot consult the ontology developers to understand its construction. One aspect of quality assurance is checking how well an ontology complies with established ‘coding standards’; is the ontology *regular* in how descriptions of different types of entities are axiomatised? Is there a similar way to describe them and are there any corner cases that are not covered by a pattern? Detection of regularities and irregularities in axiom patterns should provide ontology authors and quality inspectors with a level of abstraction such that compliance to coding standards can be automated. However, there is a lack of such reverse ontology engineering methods and tools.

**Results:**

RIO framework allows regularities to be detected in an OWL ontology, i.e. repetitive structures in the axioms of an ontology. We describe the use of standard machine learning approaches to make clusters of similar entities and generalise over their axioms to find regularities. This abstraction allows matches to, and deviations from, an ontology’s patterns to be shown. We demonstrate its usage with the inspection of three modules from SNOMED-CT, a large medical terminology, that cover “Present” and “Absent” findings, as well as “Chronic” and “Acute” findings. The module sizes are 5 065, 20 688 and 19 812 asserted axioms. They are analysed in terms of their types and number of regularities and irregularities in the asserted axioms of the ontology. The analysis showed that some modules of the terminology, which were expected to instantiate a pattern described in the SNOMED-CT technical guide, were found to have a high number of regularity deviations. A subset of these were categorised as “design defects” by verifying them with past work on the quality assurance of SNOMED-CT. These were mainly incomplete descriptions. In the worst case, the expected patterns described in the technical guide were followed by only 5% of the axioms in the module.

**Conclusion:**

It is possible to automatically detect regularities and then inspect irregularities in an ontology. We argue that RIO is a tool to find and report such matches and mismatches, for evaluations by the domain experts. We have demonstrated that standard clustering techniques from machine learning can offer a tool in the drive for quality assurance in ontologies.

**Availability:**

http://riotool.sourceforge.net/

**Contact:**

http://eleni.mikroyannidi@manchester.ac.uk, http://robert.stevens@manchehster.ac.uk

## Background

Ontologies provide an effective way for creating, using and sharing medical and biological vocabularies [1]. However, one problem with authoring ontologies is that, as they grow in size, they become more complex to both understand and maintain. An example is SNOMED-CT [2]; a leading healthcare terminology maintained across many countries [3] that have joined the International Health Care Terminology Standards Development Organisation (for a list see [2]).

Ontology construction can be based upon patterns of different abstraction level; these can be notes from the developers, general guidelines, formal documentation of the ontology, published papers describing the ontology, spreadsheets of fillers for ontology templates etc [4-6]. Such documentation or *coding standards* should offer an opportunity for quality assurance, if deviations from those guidelines can be found. The question is how does an ontology author effectively and efficiently find, not only those classes that conform to the pattern, but those classes that do not conform?

To give an example, consider the description of ’present findings’ in SNOMED-CT; these are 59 classes that have the keyword present in their label. Two example definitions of classes ’Dizziness present (situation)’, ’Paralysis present (situation)’ is shown in Additional file 1: Figure S1. Both definitions are very similar, thus a general pattern that could express them is shown in Additional file 2: Figure S2.

The expression in Additional file 2: Figure S2 has the variable ?PresentSituation, which holds all present classes and the variable ?Finding, which holds the corresponding clinical findings (represented as classes in SNOMED-CT). The expression in Additional file 2: Figure S2 is an abstraction over the *regular structural description* of present findings in the ontology. It is a *design template*, which is expected to cover most of the ‘present’ findings in SNOMED-CT. To the best of our knowledge, the detection of such patterns is done manually by the ontology engineer in the form of a query or rule.

On the other hand, deviations from this general pattern can exist in the ontology. One such deviation is shown in Additional file 3: Figure S3. The ’On examination - joint effusion present (disorder)’ even though it has the keyword ‘present’ in its label, it does not have the same definition as the classes of Additional file 1: Figure S1.

This deviation is not necessarily a design defect, but having tools for highlighting them can reveal how the ontology was built and facilitate quality assurance. The manual detection of these deviations in a large and complex ontology like SNOMED-CT is not currently feasible.

We propose the use of the Regularity Inspector for Ontologies (RIO) framework as a means of bootstrapping the quality assurance process for ontologies. We use the SNOMED-CT terminology as an example ontology to demonstrate the use of RIO: 

1. To find *regularities* (like the one in Additional file 2: Figure S2) in the use of axioms in entity description.

2. To find *deviations* from axiom patterns described in the SNOMED-CT documentation.

We argue that such a framework should facilitate the process of inspecting and reporting defects and potential defects to the domain experts for the ontology. We are interested in revealing the composition styles of the modules to an ontology engineer who is not necessarily a domain expert. Thus, they will not have to spend more time than is necessary to find ways of isolating defects in the ontology, such as the manual inspection of tangled hierarchies of classes containing many deeply nested restrictions.

### SNOMED-CT and Description Logics

This paper focuses on the Web Ontology Language (OWL) [7] representation of SNOMED-CT. OWL is based on Description Logics allowing for inferences after automated reasoning [8,9]. SNOMED-CT is deliberately based on a relatively simple variant of OWL for which computation is guaranteed to be efficient, EL++ [10], which corresponds to the OWL-EL profile [11]. This allows for two types of formulating terminology: 

1. A *stated form* that defines each concept is asserted manually by SNOMED-CT’s authors. The stated form can be also called as *asserted form*.

2. An *automated reasoner* is then used to organize the concepts logically into hierarchies based on their stated definitions. Inferences will be generated about the relationships among classes and instances.

For example, the definitions of Additional file 1: Figure S1 belong to the stated form of the ontology; they are *asserted axioms*. After automated reasoning, it will be then inferred that both ’Dizziness present (situation)’, ’Paralysis present (situation)’ are specialisations of the ’Clinical finding present (situation)’ class. This is an inference derived from the definition of these classes. On the other hand, the ’On examination - joint effusion present (disorder)’, whose definition is shown in Additional file 3: Figure S3 does not imply a ’Clinical finding present (situation)’ after reasoning.

Quality assurance of SNOMED-CT is done in two levels; on the asserted axioms of the ontology and on the inferences. The Unified Medical Language System maintains a “Core Problem List Subset”^a^ consisting of around 9 000 concepts. New technologies make it possible to identify all other concepts that affect the classification of members of the subset consisting of fewer than 40 000 concepts. The “Core Problem List Subset” includes potential design errors in the concepts of the ontology. These are mainly wrong descriptions of the concepts, resulting in wrong inferences. It should be noted that these errors are not logically wrong; the reasoner will not find any unsatisfiable concepts [12]. But in terms of semantic meaning mapped to the domain, they are incorrect (e.g. an injury of the pelvis is implied to be an injury of the foot [13]).

There are many efforts towards methods for quality assurance in SNOMED-CT [3,14-17]. However, these mainly focus on how to find modelling defects in the ontology from a domain expert’s or ontological perspective, rather than adherence to a ‘house-style’ or guidelines. Such guidelines may or may not be good modelling practise, but conforming to an ontology’s own guidelines is one aspect of quality assurance.

In the following we focus on methods that can be used for the quality assurance of the asserted form of SNOMED-CT. These methods are centered on the detection of repetitive structures in the axioms of an ontology, named as *syntactic regularities*. What is ‘in’ and what is ‘out’ of those patterns should give ontology developers a means of checking their ontology and, eventually, be an aid to ontology comprehension—that is, understanding how an ontology has been authored.

### Syntactic regularities

The existence of patterns gives rise to syntactic regularities like the one described in Additional file 1: Figure S1. These are axioms of similar syntax [18]. All the regularities that can exist in an ontology do not necessarily highlight a corresponding ‘design’ pattern. However, the recognition of syntactic regularities should be helpful for understanding the composition of the ontology, as it should reveal parts of the ontology that were designed in similar ways. This should enable the user to complete tasks, such as the extension of the ontology, its integration with other ontologies, quality assurance and so on.

Syntactic regularities can be inspected manually by writing a query or a rule. A way to do this is with OPPL (http://oppl2.sourceforge.net); which is a scripting language for manipulating ontologies [19]. For example, the pattern of Additional file 2: Figure S2 can be expressed as shown in Additional file 4: Figure S4 for obtaining the axioms that instantiate it.

OPPL has been used for quality assurance of SNOMED-CT [17] and for manual analysis of patterns in ontologies [20]. However, the ontology engineer has to inspect the ontology first and then manually write the query to check if a pattern is used.

The Regularity Inspector for Ontologies (RIO) is a framework that detects regularities based on unsupervised cluster analysis. In this paper we describe a refinement of the framework from [18] and we demonstrate its usage with three modules [21] from SNOMED-CT. We wanted to detect and further analyse the regularities and irregularities in the modules of the ontology, and find how these can be linked to potential design defects in the ontology that have been reported in past work. The assumption made for these defects is that entities that follow naming conventions should also follow a similar pattern in the description of their usage axioms. For example, any concept in the ontology that is labeled as Chronic, should also have an explicit or implicit reference to the ‘Chronic (qualifier value)’ class. Entities that do not follow this pattern are categorised as: 

1. Design discrepancies in the asserted axioms in an ontology.

2. Deliberate deviations of a pattern.

We pinpoint such defects in the ontology and we verify a portion of them by referring to the SNOMED-CT literature. The design discrepancies we highlight mainly refer to missing restrictions. The rest are categorised as deviations from an expected pattern. We show that parts of the ontology that do not follow a particular pattern are more prone to design discrepancies, such as missing restrictions, incorrect descriptions etc. That is expected, since the developers have a higher level of freedom to describe concepts that do not have a general pattern, and, therefore, there is more room for error.

## Materials and methods

### The RIO framework

In [18] we introduced RIO; a framework for spotting regularities in ontologies. The framework is based on cluster analysis, the purpose of which is to partition data into groups (clusters) that are meaningful, useful or both [22]. The RIO framework enables the partitioning of a set of entities in an ontology according to similar usage, i.e.: entities in the same cluster occur with *similar axioms* playing *similar roles*. Therefore, the detection of regularities is based on the following two general steps: 

1. The computation of clusters of similar entities in the ontology.

2. The provision of a synthetic view of all the axioms that contribute to generate a cluster of entities.

The first step is completed using hierarchical agglomerative cluster analysis [22,23]. The second is accomplished via *generalisations*, which capture the potential *syntactic regularities* in the ontology. To give an example of RIO’s goal, the expression in Additional file 2: Figure S2 is a generalisation expressing the regularity of the axioms in Additional file 1: Figure S1. Cluster analysis helps to generate groups of similar concepts, which are denoted as variables in the generalisation. For example, in the generalisation in Additional file 2: Figure S2, variable ?PresentSituation would represent a cluster that holds present finding classes.

Algorithm ?? shows the steps that are followed for the computation of clusters in an ontology. Details about the algorithm were previously reported in [18].

### Algorithm 1. RIO algorithm

In algorithm ??, steps 7-13 are common steps of agglomerative hierarchical clustering [22] and are not discussed further.

Steps 1-6 in the algorithm are the most important ones in the implementation of the framework. Cluster analysis relies on the notion of distance to quantify how similar (or dissimilar) and, therefore, how close or far apart two entities are in the clustering space. In our implementation, we compute the distance between pairs of entities based on their axiom usage. These axioms are transformed into more abstract forms using a placeholder replacement function *ϕ*, which is based on a heuristic approach. The placeholder replacement function *ϕ* enables the comparison between pairs of entities and the control of the granularity of the distance.

### Placeholder replacement function

The placeholder replacement function is applied to the axioms used in an entity’s description and decides when an entity should be replaced by a placeholder. More formally, given an ontology O, we define *Φ*={?owlClass,?owlObjectProperty,?owlDataProperty,?owlAnnotationProperty,?owlIndividual,?*} a set of six symbols that do not appear in the signature^b^ of O - sig(O). A placeholder replacement is a function ϕ:sig(O)→sig(O)∪Φ satisfying the following constraints: Consider an entity e∈O then *ϕ*(*e*)= 

•*e* or ?* or ?owlClass if *e* is a class name;

•*e* or ?* or ?owlObjectProperty if *e* is an object property name;

•*e* or ?* or ?owlDataProperty if *e* is a data property name;

•*e* or ?* or ?owlAnnotationProperty if *e* is an annotation property name;

•*e* or ?* or ?owlIndividual if *e* is an individual name.

The notion behind the placeholder replacement function *ϕ* is that we want to abstract axioms in order to capture their general structure and to calculate the distance between their referencing entities. Changing the granularity of the placeholder replacement function produces more or less sensitive distance functions. Different approaches can be adopted, such as: 

1. Naive approaches

2. Popularity replacement

3. Structural replacement

The *naive approaches* covers two extremes; replacing every entity with a placeholder or not replacing any at all. Whilst the former produces a distance that is far too tolerant and puts together entities that seem unrelated, the latter will most likely result in a distance that scores 1 (maximal distance) for most entity pairs.

In [18] we proposed a trade off where we delegated the decision of whether to replace an entity in an axiom to a measure of its *popularity* with respect to the other entities in the same kind of axiom within the ontology. We regarded entities that are used frequently in the axioms of the ontology to be more important, thus will not be replaced by the function *ϕ*. This approach seemed to work adequately for smaller and medium sized ontologies. However, for bigger ontologies this replacement policy could overfit the data; the increased popularity of many entities can result in a very sensitive distance, thus very fine-grained regularities.

#### Structural replacement function

In this paper we define a *structural replacement* function. This approach is also heuristic and is based on the search of an optimal split of the entities in a corresponding placeholder as a preprocess of the clustering. The replacement function, applied in steps 3 and 4 of algorithm ??), works as follows: 

1. A structural variable replacement is applied in the axioms of the ontology. This will create an abstraction over different types of asserted axioms in the ontology.

2. The variable replacements are refined by examining better separation of instantiated axioms in different groups.

We will demonstrate how this transformation function works in practice by using the example ontology in Additional file 5: Figure S5. This ontology is just an example to demonstrate how a particular replacement function works. It has no relationship with SNOMED-CT. 

**Step 1:** The first step is the representation of axioms in abstract forms; This is done by replacing every entity in an axiom with a general variable based on the type of the entity. Additional file 6: Figure S6 shows the transformation result for the example ontology. It should be noted that this abstraction over the axioms is different from the final generalisation which represent the detected syntactic regularities. This transformation is an intermediate step of algorithm ??.

**Figure 1:**
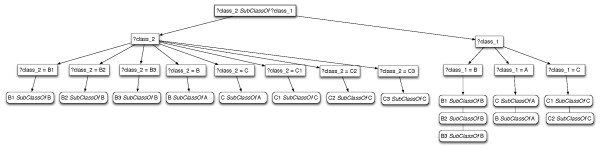
Tree showing possible variable replacements.

**Step 2:** For each one of the general axioms (14)-(16) we retrieve their instantiations and check if a replacement of a variable with an entity gives better separation of axioms in different groups. The examination of variable replacements depends on the structural commonalities of the axioms. Our criterion is that if there are more than two structural differences between a pair of axioms then the variable should be checked for further replacements. The idea behind this criterion is that we want to find an optimal variable replacement in the axioms that will reflect the differences between the entities in the ontology. In the example ontology in Additional file 6: Figure S6, the general axiom (14) abstracts the axioms (1)-(8) in Additional file 5: Figure S5. Many of these axioms have more than one structural difference (1) and (5) or (2) and (14) etc.). Therefore, further possible replacements should be examined. The general axiom is the root of the tree. Then, the branches of the tree show all possible values for each variable of the general axiom. An example tree for the generalisation (14) is shown in Figure 1. The leaf nodes of the tree show the instantiations that result from the replacement of the parent node. Replacements that abstract only a single axiom are discarded. Replacements that separate the values of the other variables into different sets and abstract more than one axiom are kept. For example, in Additional file 6: Figure S6 all further splits of variable ?class_2 are discarded as they abstract only a single axiom. However, the replacements for ?class_1 are kept as they abstract more than one axiom. Therefore, classes A, B and C in the axioms of the form of (14) are marked as “relevant” and they are not replaced by a placeholder. The same procedure is followed for the general axioms (15) and (16). In particular, none of the referenced entities of the general axioms (15) and (16) are marked as “relevant” because none of the possible replacements abstracts more than one axiom. Thus, all of the referenced entities will be replaced by a placeholder after the application of the replacement function *ϕ*.

At this point we have demonstrated how the replacement function *ϕ* works. The next step in algorithm ?? is the computation of pairwise distances (step 5). For example, the distance *d*(B1, B3)=0 as the application of *ϕ* on their referencing axioms will return the same set of transformed (see Additional file 7: Figure S7).

Finally, steps 7-13 of Algorithm ?? describe the computation of the clusters. We should comment that we selected our stopping criteria according to the maximal difference between pairs of entities. It is $\forall O,\phi ,e_{1},e_{2}:0\leq d_{\phi }(e_{1},e_{2})\leq 1$. Therefore, the algorithm will stop agglomerations when the distances between all possible pairs of elements for all clusters is equal to 1 (meaning that these entities are completely dissimilar).

### Syntactic regularities expressed with generalisations

Finally, the description of the clusters is given by *generalisations*. Generalisations provide a synthetic view of all the axioms that contribute to generate a cluster of entities. In practice they are axioms including variables that hold similar entities. Each of these axioms can be regarded as an *instantiation* of a generalisation, as they can be obtained by replacing each variable in the generalisation with entities in the signature of the ontology. Additional file 8: Figure S8 shows the generalisations and instantiations of our example ontology. The first generalisation instantiates axioms (1)-(3). The second generalisation instantiates axioms (9), (11), (13). The instantiations of the third generalisation are axioms (10), (12). Finally, the instantiations of the fourth generalisation are axioms (6)-(8) and of fifth generalisation are axioms (4), (5). In RIO, the vocabulary from OPPL [19] is used for expressing the variables in the generalisations. We also use OPPL scripts as an additional verification of the results we find.

### SNOMED-CT modules

The steps that were followed for the analysis of the regularities in SNOMED-CT were: 

1. Extraction of three SNOMED-CT modules

2. Application of the RIO on the asserted ontologies

3. Analysis of the regularities and verification with published work and SNOMED-CT documentation.

For the extraction of the modules we used the July 31 2010 IHTSDO (International Health Terminology Standards Development Organisation) release of SNOMED CT converted to OWL using the Perl script provided with the release.

The procedure for extracting the modules from the ontology is the same as the one described in [17]. For module extraction, we used the methods in the OWL API [24] (http://owlapi.sourceforge.net) packaged in an application, which is available online (http://owl.cs.manchester.ac.uk/research/topics/snomed/).

The analysis of syntactic regularities mainly focuses on the description of four groups of terms in the ontology. These groups of terms have a naming convention and it is also expected to instantiate a pattern. In the remainder of the paper they will be called target entities. We also will refer to terms in the ontology by using their labels as they were found in the OWL ontology. The extracted modules refer to “chronic” and “acute” diseases, together with “present” and “absent” clinical findings. Some metrics describing the modules are presented in Table 1.

**Table 1:** General metrics on the three extracted modules of SNOMED-CT

		**Present and absent**	**Chronic findings**	**Acute findings**
		**clinical findings**		
Target entities		Classes whose labels	Classes whose labels	Classes whose labels
		have the keywords	have the keywords	have the keywords
		“present” or “absent”	“chronic”	“acute”
Axioms		5 065	20 688	19 812
Classes		1 687	6 842	6 599
Object properties		16	25	25
Mean class hierarchy depth		9.76	11.2	10.09

The extraction of the modules was based on a set of terms that were expected to instantiate a pattern in their axioms. For example, it is expected that all chronic findings to have an axiom that relates them with ’Chronic (qualifier value)’. Similarly all acute findings are expected to be related with ’Acute (qualifier value)’. The modules describe classes that are findings and have the words “Present” and “Absent” in the beginning or middle of their label. These can be gathered using the OPPL script in Additional file 9: Figure S9:

The OPPL script gives 0 candidate classes whose name starts with the word “Present” and 59 classes with the word “present” in the middle of their name. For the “absent” case, there was only 1 class whose label started with the word “Absent” and 24 classes with the word “absent in the middle of their name.

Similarly, the “Acute” and “Chronic” modules were extracted based on a set of terms which have the words “Acute” and “Chronic” in their name and describe acute and chronic clinical findings respectively. Similar scripts as the ones presented in Additional file 9: Figure S9 gave 420 candidate “Chronic” classes and 509 “Acute” candidate classes.

In our analysis the patterns which are described in the technical guide are manually formulated using OPPL scripts, which returns all possible candidate axioms that are instantiations of this pattern. These are compared with RIO’s results and we further discuss the strengths and weaknesses of the two methods.

## Results

Table 2 shows metrics of the application of the RIO framework on each module. There are two main metrics we use in order to measure the regularity of an ontology: 

1. Mean cluster coverage per generalisation;

2. Mean number of instantiations per generalisation.

**Table 2:** Results of the application of the RIO framework in the three SNOMED-CT modules

**Module**		**# Clusters**	**Cluster coverage per**	**Mean Instantiations**
			**generalisation (%)**	**per Generalisation**
Present and absent clinical findings		41	8.50	6.42
Chronic findings		75	6.40	6.13
Acute findings		76	6.80	5.70

The mean cluster coverage per generalisation shows the level of coverage of a cluster by a single generalisation. In principle, the variable of a generalisation does not necessarily hold all members of a cluster, but a subset of them. For example, the third generalisation in Additional file 8: Figure S8 has two instantiations and covers 66% of entities of cluster_2_ and 50% of entities of cluster_1_.

The mean number of instantiations per generalisation shows how many axioms can be abstracted by a single generalisation. An ontology with a high cluster coverage per generalisation (close to 100%) and a high mean of instantiations per generalisation is a strong indication of a very regular ontology.

The results show that the present and absent clinical findings module is the most regular of all three modules, as it has the highest cluster coverage and mean instantiations per generalisations. However, the overall cluster coverage percentage does not exceed 8.5%. There are two reasons for this result; First, the ontology is not highly regular. Secondly, in some cases the clustering algorithm is too “greedy”, resulting in big clusters that are not completely homogeneous.

Most of the regularities that were captured by RIO refer to restrictions using the RoleGroup attribute [25] for grouping relationships. This is also the main regularity that is described in the technical guide of the release we used [26]. The purpose of the RoleGroup attribute in SNOMED-CT was to provide a simple way to indicate that certain roles should be grouped together [25]. However, we want to check how this general regularity is formed when describing different sets of terms in the ontology. In the remainder of the section, we will focus on the regularities we found in the entities from the ontology, which have: 

•The words “Present” or “Absent” at the beginning or in the middle of their name.

•The words “Chronic” or “Acute” at the beginning or in the middle of their name.

### “Present” and “absent” cases

Table 3 shows the results of the regularities referring to entities, whose names included the words “present” or “absent”. There are 58 out of 59 “present” entities which are distributed in 6 clusters. Similarly, all the 25 “absent” entities are distributed in 5 clusters.

**Table 3:** Selected results of the analysis of regularities in present and absent cases

	**Present**	**Absent**
Total number of entities starting with “Present” or “Absent”	0	1
Total number of entities having “present” or “absent” in the middle of their name	59	24
Number of clusters that include the target entities	6	5
Number of generalisations describing the target entities	65	39
Number of instantiations referring to the target entities	404	236
Number of target entities that were not in any cluster.	1	0
Number of clusters including entities with multiple role groups (RoleGroup) in their axioms	3	3
Number of clustered entities using multiple role groups (RoleGroup) in their axioms	4	3
Number of generalisations which instantiations explicitly refer to the present (Known present (qualifier value)) or absent qualifier (Known absent (qualifier value))	23 (35%)	15 (39%)
Number of instantiations that explicitly refer to the present or absent qualifier	127 (31%)	81 (34%)

There is one “present” clinical finding that is not included in the clusters (‘Definitely present (qualifier value)’). The reason is that this class is never used in any other axiom, apart from its declaration and position in the class hierarchy (It is a subclass of the ‘Known present (qualifier value)’ and superclass of the ‘Confirmed present (qualifier value)’. Fifty of the “present” entities appear to be in the same cluster and described by 16 generalisations. Additional file 10: Figure S10 shows an example generalisation and instantiation referring to this cluster (cluster_1_).

The structure of the 16 generalisations describing “present” classes is similar; in many cases the only thing that changes is a single variable in the generalisation. A reason for this is that entities participating in axioms of similar syntax fall into different clusters due to their different usage in other axioms. The expected pattern for all the present and absent cases is their explicit reference to the ’Known present (qualifier value)’ and ’Known absent (qualifier value)’ respectively. An example instantiation pattern is shown in Additional file 10: Figure S10.

The analysis of the regularities (Table 3) showed that the 31% (127) of the usage axioms of the present entities are using this pattern. These axioms are abstracted by 23 (35%) of the generalisations. Similarly, for the absent classes, 81 (34%) instantiations explicitly refer to the absent qualifier value and these are abstracted by 15 (39%) by the generalisations. Note that the total number of instantiations in Table 3 refer to all the axioms that were generalised and reference by at least one target entity (including both left and right hand side of the axiom). Also these numbers refer to the expected syntactic pattern, which is an explicit reference to a qualifier value (e.g. ’Known present (qualifier value)’). However, an implicit pattern can exist, such as propagation through the class hierarchy, which can infer such a connection. This explains the relatively low percentage of the axioms following the pattern.

Similarly, 21 of the “absent” clinical findings are in the same cluster described by 10 generalisations. Both *absent* and *present* classes seem to follow the same type of regularity in their definition. This is also described as a common pattern in the SNOMED-CT online resources [26]. Most of the entities are described using the RoleGroup attribute for grouping relationships using the ‘Associated finding (attribute)’, ‘Finding context (attribute)’, ‘Temporal context (attribute)’ and ‘Subject relationship context (attribute) attributes. However, in both cases, we found deviations from this pattern. For example the class ‘On examination - joint effusion present (disorder)’ belongs to a different cluster as it is defined differently (Additional file 3: Figure S3).

There were also clusters including entities with different forms of class expressions. An example is the ‘Clinical finding absent (situation)’. The analysis of the module gave in total 17 classes that included the ’RoleGroup’ attribute more than once in their definition. Additional file 11: Figure S11 shows three example clusters with such entities. The usage of role groups and multiple role groups in SNOMED-CT is described in [27].

These examples might be deliberately defined in this way, however it is a deviation from the design style of most classes, that leads to deeply nested class expressions. It might be also a case of malformed axioms, as it is not clear in which cases the ’RoleGroup’ attribute should be used more than once in the same axiom [27] and when a relationship should be grouped with an existing role group. Table 3 shows that 3 clusters were detected with 4 “present” classes using multiple role groups in their axioms. Likewise, 3 clusters were detected with 3 “absent” classes whose axioms used multiple role groups. Our aim is to highlight such cases, which should be further assessed by experts.

### “Chronic” and “Acute” cases

Table 4 summarises the results of the regularities that were found in the entities containing the words “acute” and “chronic” in their label. The results showed that most of the entities do not follow a general pattern. Therefore, the entities are distributed in many clusters and are described by many generalisations. From the technical guide, a general pattern that is expected in these terms is the explicit reference to the chronic or acute qualifiers in equivalent or subclass axioms [17]. An example description is shown in Additional file 12: Figure S12.

**Table 4:** Selected results on the analysis of regularities in chronic and acute cases

	**Chronic**	**Acute**
Total number of entities starting with “Chronic” or “Acute”	388	472
Total number of entities having “chronic” or “acute” in the middle of their name	32	38
Number of clusters that include the target entities	34	34
Number of generalisations describing the target entities	919	1109
Number of instantiations referring to the target entities	1503	1849
Number of target entities that were not in any cluster.	12	11
Number of clusters including entities with multiple role groups (RoleGroup) in their axioms	19	21
Number of clustered entities using multiple role groups (RoleGroup) in their axioms	64	79
Number of generalisations whose instantiations explicitly refer to the chronic (Chronic (qualifier value)) or acute qualifier	50 (5%)	114 (10%)
Number of instantiations that explicitly refer to the chronic or acute qualifier	76 (5%)	210 (11%)

However, only 50(5%) of the generalisations for the “Chronic” module were found to abstract axioms related to “Chronic” entities and 114(10%) of the generalisations for the “Acute” module abstracted axioms related to “Acute” entities. An example generalisation reflecting the expected pattern for the chronic classes is shown in Additional file 13: Figure S13.

We verified some of the results of this work with the results described in [17]. However, this paper focuses more on the analysis of the syntactic regularities from an ontology engineering perspective. Subsets of these terms might be expected to deviate from this pattern from a medical perspective. For example, a subset of “chronic” terms deviate from the pattern in Additional file 13: Figure S13 reported in [17], as they are described according to their morphology. Thus, there is no existential restriction in their asserted axioms referring to the ’Chronic (qualifier value)’. Since these terms do not have a reference to the Chronic (qualifier value) they cannot be highlighted by a syntactic tool like RIO; such a relationship is found in the inferences of the ontology.

In addition, 20 entities from the target set of entities were not clustered. Here, for the sake of brevity, we mainly focus on the “Chronic” cases. Table 4 summarises some of the results for all the cases. Additional file 15: Figure S15 shows the chronic entities, which were not included in any cluster. Some of them, such as the ‘Chronic anxiety (finding)’ were also reported in [17] as design defects. From these, the “Chronic low back pain (finding)”, is reported as having an incomplete description in [17]. In particular, the class has an existential restriction that is missing. However, this type of irregularity is not clear from the syntax of the axiom. The class is grouped with other “chronic” classes, which have complete existential restrictions. Therefore, this type of irregularity is not easily noticeable from the analysis of the results.

Finally, 10 clusters included entities participating in nested class expressions with multiple role groups (using multiple RoleGroup relationships). It should be noted that clusters of smaller size tended to include such deviations from the regular pattern (most of the class expressions are described using a single role group in the examined modules).

### Verification of results

In order to verify some of the results, we manually ran OPPL scripts whenever possible. In particular, we examined, which classes failed to follow the expected pattern by running corresponding OPPL scripts. For example, for the “chronic” classes we ran the queries of Additional file 16: Figure S16 to select classes that had the word “Chronic” in their label, but were missing the expected semantics. The first OPPL script of Additional file 16: Figure S16 gave 131 candidate classes with incomplete semantics while the second script will give 5 candidate classes with incomplete semantics. This set of candidate classes are potential errors in the ontology since they are missing the reason for being “chronic” findings—despite this is indicated in their label.

Comparing the results of the manual analysis using OPPL scripts with the results of the automatic analysis by RIO we can note that the analysis with RIO gave in total 314 classes (Table 4) as potential deviations from the expected pattern while manual OPPL scripts narrowed this down to 131 candidate classes. The reason for this difference is that OPPL scripts take into account both the asserted and the inferred form of the ontology, thus the instantiation of the expected pattern is in the inferences of the ontology. However, this kind of semantic analysis could not be done by RIO since we have a purely syntactic approach. It should be mentioned, though, that the 131 candidate classes from the OPPL script included all these classes in Additional file 15: Figure S15. Similar OPPL scripts gave 147 “acute”, 9 “present” and 2 “absent” candidate classes with missing descriptions.

## Conclusions and future work

We have presented a refinement of RIO [18]; a framework that detects syntactic regularities in an ontology using cluster analysis, and applied it to three modules from SNOMED-CT. In particular, we have presented a different transformation function for the calculation of the distance that is used for the clustering. This transformation function finds optimal representations of entities by placeholders according to structural differences in axioms. The framework allows the use of different transformation functions, without affecting the implementation of the remaining steps in the workflow; thus, future work could include testing of other options. There may be better transformation policies; what we have done here is to show that clustering is possible and that it can be used to spot regularities and irregularities in patterns of axioms within an ontology.

We demonstrated the use of RIO with three modules from SNOMED-CT. The modules were selected according to a set of entities which were expected to follow a pattern that also matches their label. Our aim was to inspect the regularities and irregularities that might reveal the composition style of the ontology to the ontology engineer, who may not be a domain expert. Some of the results are confirmed through documentation of the ontology or past publications related to quality assurance methods for SNOMED-CT. In addition, OPPL scripts were used for expressing expected patterns and comparing the results with the ones from RIO. The results showed expected regularities, as well as deviations from these regularities. It revealed terms with incomplete descriptions, such as missing existential restrictions (e.g. 12 “chronic” classes with an incomplete description which were not included in any cluster); classes, which were placed correctly in the class hierarchy by the reasoner, but described with long class expressions using multiple role groups (e.g. 79 “acute” classes whose definition makes use of multiple role groups). In the worst case, the expected patterns described in the technical guide of the ontology was explicitly instantiated by only 5% of the corresponding entities in the module (Table 4). The results also indicated that parts of the ontology that did not follow an explicit pattern tended to have more potential “defects”. All these can be detected and reported to domain experts who will decide which ones should be modified.

In addition, with OPPL scripts (Additional file 16: Figure S16) we verified a subset of entities which were also highlighted with the analysis from RIO (e.g. entities of Additional file 15: Figure S15). However, a number of target entities did not instantiate the expected pattern in their asserted axioms, but the pattern was expressed in the inferences of the ontology. These entities were highlighted by RIO as deviations from the expected pattern as the inferences were not taken into account. The consideration of inferences by RIO for detecting patterns in both asserted and inferred ontology is future work.

There were frequently generalisations expressing the same type of regularity. A future development could be to check possible bindings of such generalisations into generalisations that will give a more uniform representation of the regularity.

We argue that RIO should be useful as an intermediate step of a more systematic quality assurance procedure for ontologies. Our use of a basic clustering approach has a demonstrable use in finding regularities and irregularities in an ontology. By itself RIO does not find defects, it only finds syntactic regularities and irregularities; it is for the ontology engineer and domain expert to determine whether or not the deviations are legitimate. It has potential for offering authors a means to gain generalisations of the major portions of an ontology; to detect deviations from a given style of representation and to facilitate the quality assurance of complex ontologies such as SNOMED-CT and many others.

## Endnotes

^a^http://www.nlm.nih.gov/research/umls/Snomed/core_subset.html.^b^For signature here we mean the set of class names, data/object/annotation property names, individuals referenced in the axioms of an ontology O.

## Competing interests

The authors declare that they have no competing interests.

## Authors’ contributions

EM led this effort, working in the core ideas about the RIO framework and in the implementations for the RIO and methods used for quality assurance. RS provided expertise in KR and in general aspects of quality assurance in ontologies. LI has contributed on the methods and implementation of the RIO framework and in the implementation of OPPL. AR provided expertise in the SNOMED ontology and verification of the quality assurance results. All authors read and approved the final manuscript.

## Supplementary Material

Additional file 1 **Figure S1.** Two axioms defining the ’Dizziness present (situation)’ and ’Paralysis present (situation)’ in SNOMED-CT. The structure of these axioms is very similar. Click here for file

Additional file 2 **Figure S2.** An example pattern for describing ’present’ clinical findings (e.g. ’Paralysis present (situation)’ and ’Dizziness present (situation)’). This pattern contains variables (?PresentSituation, ?Finding), which hold classes of similar axiom usage. Click here for file

Additional file 3 **Figure S3.** The definition of a present disorder (’On examination - joint effusion present (disorder)’). Its definition deviates from the pattern of Additional file 2: Figure S2. Click here for file

Additional file 4 **Figure S4.** Example OPPL script for detecting instances of a pattern. It defines two class variables; ?PresentSituation, ?Finding. The SELECT statement will select all axioms that instantiate this variable expression. The ADD statement will add all entities that instantiate the SELECT statement as subclasses of the PatternInstance class. Click here for file

Additional file 5 **Figure S5.** Example ontology. Click here for file

Additional file 6 **Figure S6.** Result of step 1 of the replacement procedure. The transformation of the axioms of Additional file 5: Figure S5 is shown. Click here for file

Additional file 7 **Figure S7.** Reason for *d*(B1, B3)=0. Both entities have the same set of transformed axioms shown in this figure. Click here for file

Additional file 8 **Figure S8.** Clusters, generalisations and covered instantiations for the example ontology. Click here for file

Additional file 9 **Figure S9.** OPPL scripts for gathering target terms for the extraction of the “Present” module. Click here for file

Additional file 10 **Figure S10.** Example generalisation and instantiation for ?cluster_1_. The cluster includes 50 classes with the word “present” in their name described by 16 generalisations. The example generalisation and instantiation show the pattern that is used for describing these entities, which is the usage of particular roles. Click here for file

Additional file 11 **Figure S11.** Outlier clusters with multiple usage of the RoleGroup attribute and example instantiation. Click here for file

Additional file 12 **Figure S12.** Example description of a “chronic” class (’Chronic urate nephropathy (disorder)’). Click here for file

Additional file 13 **Figure S13.** Example syntactic regularity that covers 14 axioms describing 14 chronic disorders. This syntactic regularity reflects a pattern that expected to be found for “chronic” classes (explicit reference to the ’Chronic (qualifier value)’). Click here for file

Additional file 14 **Figure S14.** Chronic entities that were not included in a cluster. Click here for file

Additional file 15 **Figure S15.** Absent entities that were not included in a cluster. Click here for file

Additional file 16 **Figure S16.** OPPL scripts for gathering chronic classes with incomplete description. Click here for file
