# Supplementary material for: Analysing Syntactic Regularities and Irregularities in SNOMED-CT
Source: J Biomed Semantics. 2012 Dec 17;3:8. doi: 10.1186/2041-1480-3-8 (PMC3637289; doi:10.1186/2041-1480-3-8)
Supplement: Additional file 1 — Figure S1. Two axioms defining the ’Dizziness present (situation)’ and ’Paralysis present (situation)’ in SNOMED-CT. The structure of these axioms is very similar. [file 2041-1480-3-8-S1.pdf]

'Dizziness present (situation)' *EquivalentTo*
'Clinical finding present (situation)'
and (RoleGroup **some**
(('Associated finding (attribute)' **some** 'Dizziness (finding)')
and '(Finding context (attribute)' **some** 'Known present (qualifier value)')
and '(Temporal context (attribute)' **some** 'Current or specified time (qualifier value)')
and ('Subject relationship context (attribute)' **some** 'Subject of record (person)')))

'Paralysis present (situation)' *EquivalentTo*
'Clinical finding present (situation)'
and (RoleGroup **some**
(('Associated finding (attribute)' **some** 'Paralysis (finding)')
and ('Finding context (attribute)' **some** 'Known present (qualifier value)')
and ('Temporal context (attribute)' **some** 'Current or specified time (qualifier value)')
and ('Subject relationship context (attribute)' **some** 'Subject of record (person)')))
