# Supplementary material for: Analysing Syntactic Regularities and Irregularities in SNOMED-CT
Source: J Biomed Semantics. 2012 Dec 17;3:8. doi: 10.1186/2041-1480-3-8 (PMC3637289; doi:10.1186/2041-1480-3-8)
Supplement: Additional file 2 — Figure S2. An example pattern for describing ’present’ clinical findings (e.g. ’Paralysis present (situation)’ and ’Dizziness present (situation)’). This pattern contains variables (?PresentSituation, ?Finding), which hold classes of similar axiom usage. [file 2041-1480-3-8-S2.pdf]

?PresentSituation *EquivalentTo*
'Clinical finding present (situation)'
and (RoleGroup **some**
(('Associated finding (attribute)' **some** ?Finding)
and ('Finding context (attribute)' **some** 'Known present (qualifier value)')
and ('Temporal context (attribute)' **some** 'Current or specified time (qualifier value)')
and ('Subject relationship context (attribute)' **some** 'Subject of record (person)')))

where:
?PresentSituation = ['Paralysis present (situation)', 'Dizziness present (situation)'...]
?Finding = ['Paralysis (finding)', 'Dizziness (finding)'...]
