# Supplementary material for: Analysing Syntactic Regularities and Irregularities in SNOMED-CT
Source: J Biomed Semantics. 2012 Dec 17;3:8. doi: 10.1186/2041-1480-3-8 (PMC3637289; doi:10.1186/2041-1480-3-8)
Supplement: Additional file 3 — Figure S3. The definition of a present disorder (’On examination - joint effusion present (disorder)’). Its definition deviates from the pattern of Additional file 2: Figure S2. [file 2041-1480-3-8-S3.pdf]

'On examination - joint effusion present (disorder)' *EquivalentTo*
'On examination - specified examination finding (finding)'
and 'Effusion of joint (disorder)'
