# Supplementary material for: Analysing Syntactic Regularities and Irregularities in SNOMED-CT
Source: J Biomed Semantics. 2012 Dec 17;3:8. doi: 10.1186/2041-1480-3-8 (PMC3637289; doi:10.1186/2041-1480-3-8)
Supplement: Additional file 4 — Figure S4. Example OPPL script for detecting instances of a pattern. It defines two class variables; ?PresentSituation, ?Finding. The SELECT statement will select all axioms that instantiate this variable expression. The ADD statement will add all entities that instantiate the SELECT statement as subclasses of the PatternInstance class. [file 2041-1480-3-8-S4.pdf]

```
?PresentSituation:CLASS, ?Finding:CLASS
SELECT ?PresentSituation EquivalentTo
'Clinical finding present (situation)'
  and (RoleGroup some
    (('Associated finding (attribute)' some ?Finding)
    and ('Finding context (attribute)' some 'Known present (qualifier value)')
    and ('Temporal context (attribute)' some
       'Current or specified time (qualifier value)')
    and ('Subject relationship context (attribute)'
       some 'Subject of record (person)')))
 ADD ?PresentSituation SubClassOf PatternInstance
 END;
```
