# Supplementary material for: Analysing Syntactic Regularities and Irregularities in SNOMED-CT
Source: J Biomed Semantics. 2012 Dec 17;3:8. doi: 10.1186/2041-1480-3-8 (PMC3637289; doi:10.1186/2041-1480-3-8)
Supplement: Additional file 5 — Figure S5. Example ontology. [file 2041-1480-3-8-S5.pdf]

B1 *SubClassOf* B (1)

B2 *SubClassOf* B (2)

B3 *SubClassOf* B (3)

C *SubClassOf* A (4)

B *SubClassOf* A (5)

C1 *SubClassOf* C (6)

C2 *SubClassOf* C (7)

C3 *SubClassOf* C (8)

C3 *SubClassOf* hasP1 **some** B3 (9)

C3 *SubClassOf* hasP1 **only** B3 (10)

C4 *SubClassOf* hasP1 **some** B4 (11)

C4 *SubClassOf* hasP1 **only** B4 (12)

C5 *SubClassOf* hasP1 **some** B1 (13)
