# Supplementary material for: Analysing Syntactic Regularities and Irregularities in SNOMED-CT
Source: J Biomed Semantics. 2012 Dec 17;3:8. doi: 10.1186/2041-1480-3-8 (PMC3637289; doi:10.1186/2041-1480-3-8)
Supplement: Additional file 6 — Figure S6. Result of step 1 of the replacement procedure. The transformation of the axioms of Additional file 5: Figure S5 is shown. [file 2041-1480-3-8-S6.pdf]

$$?\text{class\_2}\ \textit{SubClassOf}\ ?\text{class\_1} \tag{14}$$

$$?\text{class\_2}\ \textit{SubClassOf}\ ?\text{objectProperty\_1}\ \textbf{some}\ ?\text{class\_1} \tag{15}$$

$$?\text{class\_2}\ \textit{SubClassOf}\ ?\text{objectProperty\_1}\ \textbf{only}\ ?\text{class\_1} \tag{16}$$
