# Supplementary material for: Analysing Syntactic Regularities and Irregularities in SNOMED-CT
Source: J Biomed Semantics. 2012 Dec 17;3:8. doi: 10.1186/2041-1480-3-8 (PMC3637289; doi:10.1186/2041-1480-3-8)
Supplement: Additional file 7 — Figure S7. Reason for d(B1, B3)=0. Both entities have the same set of transformed axioms shown in this figure. [file 2041-1480-3-8-S7.pdf]

?* *SubClassOf* B

?owlClass *SubClassOf* ?owlObjectProperty **some** ?*

?owlClass *SubClassOf* ?owlObjectProperty **only** ?*
