# Supplementary material for: Analysing Syntactic Regularities and Irregularities in SNOMED-CT
Source: J Biomed Semantics. 2012 Dec 17;3:8. doi: 10.1186/2041-1480-3-8 (PMC3637289; doi:10.1186/2041-1480-3-8)
Supplement: Additional file 8 — Figure S8. Clusters, generalisations and covered instantiations for the example ontology. [file 2041-1480-3-8-S8.pdf]

Clusters:

$?\text{cluster}_1 : \{B1, B2, B3, B4\}$

$?\text{cluster}_2 : \{C3, C4, C5\}$

$?\text{cluster}_3 : \{C1, C2\}$

$?\text{cluster}_4 : \{B, C\}$

**Generalisations:**	**Instantiations:**
$?\text{cluster}_1$ *SubClassOf* B	B1 *SubClassOf* B
	B2 *SubClassOf* B
	B3 *SubClassOf* B
$?\text{cluster}_2$ *SubClassOf* hasP1 **some** $?\text{cluster}_1$	C3 *SubClassOf* hasP1 **some** B3
	C4 *SubClassOf* hasP1 **some** B4
	C5 *SubClassOf* hasP1 **some** B1
$?\text{cluster}_2$ *SubClassOf* hasP1 **only** $?\text{cluster}_1$	C3 *SubClassOf* hasP1 **only** B3
	C4 *SubClassOf* hasP1 **only** B4
$?\text{cluster}_3$ *SubClassOf* C	C1 *SubClassOf* C
	C2 *SubClassOf* C
	C3 *SubClassOf* C
$?\text{cluster}_4$ *SubClassOf* A	C *SubClassOf* A
	B *SubClassOf* A
