# Supplementary material for: Analysing Syntactic Regularities and Irregularities in SNOMED-CT
Source: J Biomed Semantics. 2012 Dec 17;3:8. doi: 10.1186/2041-1480-3-8 (PMC3637289; doi:10.1186/2041-1480-3-8)
Supplement: Additional file 9 — Figure S9. OPPL scripts for gathering target terms for the extraction of the “Present” module. [file 2041-1480-3-8-S9.pdf]

```
?c:CLASS, ?x:CONSTANT = MATCH(".Present.*")
SELECT  ?c.IRI label ?x
BEGIN
ADD ?c subClassOf PresentInTheBeginningCandidate
END;

?c:CLASS, ?x:CONSTANT = MATCH(".*present.*")
SELECT  ?c.IRI label ?x
BEGIN
ADD ?c subClassOf PresentInTheMiddleCandidate
END;
```
