# Supplementary material for: Analysing Syntactic Regularities and Irregularities in SNOMED-CT
Source: J Biomed Semantics. 2012 Dec 17;3:8. doi: 10.1186/2041-1480-3-8 (PMC3637289; doi:10.1186/2041-1480-3-8)
Supplement: Additional file 10 — Figure S10. Example generalisation and instantiation for ?cluster1. The cluster includes 50 classes with the word “present” in their name described by 16 generalisations. The example generalisation and instantiation show the pattern that is used for describing these entities, which is the usage of particular roles. [file 2041-1480-3-8-S10.pdf]

Generalisation:
?cluster$_1$ *EquivalentTo* ?cluster$_{20}$
and ?cluster$_{23}$ **some** ?expression_conjunction
and ?cluster$_{23}$ **some** ?cluster$_{16}$

Example Instantiation:
'Coin sign present (situation)' *EquivalentTo*
'Clinical finding present (situation)'
and (RoleGroup **some** ((Associated finding (attribute) **some** 'Coin sign (finding)')
and ('Finding context (attribute)' **some** 'Known present (qualifier value)')
and ('Temporal context (attribute)' **some** 'Current or specified time (qualifier value)')
and ('Subject relationship context (attribute)' **some** 'Subject of record (person)')))

where:
?cluster$_1$: CLASS=['Coin sign present (situation)'],
?cluster$_{20}$: CLASS=['Clinical finding present (situation)'],
?cluster$_{23}$: OBJECTPROPERTY=[RoleGroup, Subject relationship context (attribute)],
?expression_conjunction: CLASS=[((Associated finding (attribute) **some** Coin sign (finding))
and (Finding context (attribute) **some** Known present (qualifier value))
and (Temporal context (attribute) **some** Current or specified time (qualifier value))],
?cluster$_{16}$: CLASS=['Subject of record (person)']
