# Supplementary material for: Analysing Syntactic Regularities and Irregularities in SNOMED-CT
Source: J Biomed Semantics. 2012 Dec 17;3:8. doi: 10.1186/2041-1480-3-8 (PMC3637289; doi:10.1186/2041-1480-3-8)
Supplement: Additional file 11 — Figure S11. Outlier clusters with multiple usage of the RoleGroup attribute and example instantiation. [file 2041-1480-3-8-S11.pdf]

?cluster$_{19}$:
'Foreign body in female genital organs and perineum (disorder)'
'On examination - diabetic maculopathy absent both eyes (situation)'
'Bilateral cataracts (disorder)'
'Alexia and agraphia present (situation)'
'Foreign body of body cavity and wall (disorder)'
'Injury to heart and lung (disorder)'
'Tumor of lower respiratory tractand mediastinum (disorder)'
'On examination - diabetic maculopathy present both eyes (situation)'

?cluster$_{20}$:
'Musculoskeletal and connective tissue disorder (disorder)'
'Finding with explicit context (situation)'
'History of clinical finding in subject (situation)'
'Clinical finding present (situation)'
'Clinical finding absent (situation)'
'Disorder of soft tissue of thoracic cavity (disorder)'

?cluster$_{28}$:
'On examination - genitalia (finding)'
'On examination - skin (finding)'
'Disorder of soft tissue of body cavity (disorder)'

(a) Outlier clusters with multiple usage of RoleGroup attribute in their axioms

Clinical finding absent (situation) *EquivalentTo*
Situation with explicit context (situation) **and**
(RoleGroup **some** (Finding context (attribute)
some Known absent (qualifier value)))
and (RoleGroup **some**
(Temporal context (attribute)
some Current or specified time (qualifier value)))
and (RoleGroup **some**
(Subject relationship context (attribute)
some Subject of record (person)))

(b) Example axiom with multiple usage of the RoleGroup attribute
