# Supplementary material for: Analysing Syntactic Regularities and Irregularities in SNOMED-CT
Source: J Biomed Semantics. 2012 Dec 17;3:8. doi: 10.1186/2041-1480-3-8 (PMC3637289; doi:10.1186/2041-1480-3-8)
Supplement: Additional file 12 — Figure S12. Example description of a “chronic” class (’Chronic urate nephropathy (disorder)’). [file 2041-1480-3-8-S12.pdf]

'Chronic urate nephropathy (disorder)' *SubClassOf* 'Urate nephropathy (disorder)'
and (RoleGroup **some** ('Clinical course (attribute)' **some** 'Chronic (qualifier value)'))
