# Supplementary material for: Analysing Syntactic Regularities and Irregularities in SNOMED-CT
Source: J Biomed Semantics. 2012 Dec 17;3:8. doi: 10.1186/2041-1480-3-8 (PMC3637289; doi:10.1186/2041-1480-3-8)
Supplement: Additional file 13 — Figure S13. Example syntactic regularity that covers 14 axioms describing 14 chronic disorders. This syntactic regularity reflects a pattern that expected to be found for “chronic” classes (explicit reference to the ’Chronic (qualifier value)’). [file 2041-1480-3-8-S13.pdf]

Generalisation:
?cluster$_2$ *EquivalentTo* ?cluster$_{12}$ **and** (RoleGroup **some** (?cluster$_{32}$ **some** ?cluster$_{20}$))

Example instantiation:
'Chronic pyonephrosis (disorder)' *EquivalentTo* 'Pyonephrosis (disorder)'
and (RoleGroup **some** ('Clinical course (attribute)' **some** 'Chronic (qualifier value)'))

where:
?cluster$_2$:CLASS=['Chronic pyonephrosis (disorder)'],
?cluster$_{12}$:CLASS=['Pyonephrosis (disorder)'],
?cluster$_{32}$:OBJECTPROPERTY=[Clinical course (attribute)],
?cluster$_{40}$:CLASS=['Chronic (qualifier value)']
