# Supplementary material for: Analysing Syntactic Regularities and Irregularities in SNOMED-CT
Source: J Biomed Semantics. 2012 Dec 17;3:8. doi: 10.1186/2041-1480-3-8 (PMC3637289; doi:10.1186/2041-1480-3-8)
Supplement: Additional file 14 — Figure S14. Chronic entities that were not included in a cluster. [file 2041-1480-3-8-S14.pdf]

'Chronic progressive renal failure (disorder)',
'Chronic back pain (finding)',
'Chronic diarrhea of unknown origin (disorder)',
'Chronic inflammatory demyelinating polyneuritis (disorder)',
'Chronic cough (finding)', 'Chronic anxiety (finding)',
'Chronic post-traumatic stress disorder (disorder)',
'Chronic bullous emphysema (disorder)',
'Chronic acquired lymphedema (disorder)',
'Chronic diarrhea (disorder)', 'Chronic constipation (disorder)',
'Chronic pain syndrome (disorder)'
