# Supplementary material for: Analysing Syntactic Regularities and Irregularities in SNOMED-CT
Source: J Biomed Semantics. 2012 Dec 17;3:8. doi: 10.1186/2041-1480-3-8 (PMC3637289; doi:10.1186/2041-1480-3-8)
Supplement: Additional file 15 — Figure S15. Absent entities that were not included in a cluster. [file 2041-1480-3-8-S15.pdf]

'Acute exacerbation of bronchiectasis (disorder)',
'Acute cardiac pulmonary edema (disorder)',
'Acute coronary syndrome (disorder)',
'Acute and subacute liver necrosis (disorder)',
'Acute myeloid leukemia with recurrent genetic abnormality (morphologic abnormality)',
'Gallbladder calculus with acute cholecystitis and no obstruction (disorder)',
'Acute situational disturbance (disorder), Acute urticaria (disorder)',
'Acute meniscal tear, medial (disorder)',
'Acute contagious conjunctivitis (disorder)',
'Acute schizophrenia-like psychotic disorder (disorder)'
