# Supplementary material for: Analysing Syntactic Regularities and Irregularities in SNOMED-CT
Source: J Biomed Semantics. 2012 Dec 17;3:8. doi: 10.1186/2041-1480-3-8 (PMC3637289; doi:10.1186/2041-1480-3-8)
Supplement: Additional file 16 — Figure S16. OPPL scripts for gathering chronic classes with incomplete description. [file 2041-1480-3-8-S16.pdf]

```
1.
?c:CLASS, ?x:CONSTANT=MATCH(".Chronic.*")
SELECT  ?c.IRI label ?x
WHERE FAIL  ?c subClassOf RoleGroup some
 ('Clinical course (attribute)' some 'Chronic (qualifier value)')
BEGIN ADD ?c subClassOf ChronicIncompleteCandidates
END;

2.
?c:CLASS, ?x:CONSTANT=MATCH(".*chronic.*")
SELECT  ?c.IRI label ?x
WHERE FAIL  ?c subClassOf RoleGroup some
 ('Clinical course (attribute)' some 'Chronic (qualifier value)')
BEGIN ADD ?c subClassOf ChronicIncompleteCandidates
END;
```
